# Reproducing fear: the effect of birth stories on nulligravid women’s birth preferences

**DOI:** 10.1186/s12884-021-03944-w

**Published:** 2021-06-28

**Authors:** Yvette D. Miller, Marion Danoy-Monet

**Affiliations:** 1 School of Public Health and Social Work, Queensland University of Technology, Victoria Park Road, Kelvin Grove, QLD 4059 Australia; 2 School of Psychology and Counselling, Queensland University of Technology, Victoria Park Road, Kelvin Grove, 4059 Queensland Australia

**Keywords:** Parturition, Nulliparity, Communications media, Childbirth, Decision-making, Self-efficacy

## Abstract

**Objective:**

Preference for caesarean birth is associated with higher fear and lower self-efficacy for vaginal birth. Vicarious experience is a strong factor influencing self-efficacy in nulligravid women, and is increasingly accessible via digital and general media. This study assessed the effect of exposure to different birth stories on nulligravid women’s childbirth preferences and the factors mediating these effects.

**Methods:**

Nulligravid women (*N* = 426) were randomly allocated to one of four conditions exposing them to written birth stories. Stories varied by type of birth (vaginal/caesarean) and storyteller evaluation (positive/negative) in a 2 × 2 design. Childbirth preference, fear of labour and vaginal birth, and self-efficacy for vaginal birth were measured before and after exposure via a two-way between groups analysis of covariance. Hierarchical regression models were used to determine the mediating effects of change in childbirth fear and childbirth self-efficacy.

**Results:**

Variations in type of birth and storyteller evaluation significantly influenced childbirth preferences (*F* (1, 421) = 44.78, *p* < 0.001). The effect of vaginal birth stories on preference was significantly mediated by fear of labour and vaginal birth and self-efficacy. Effects of exposure to caesarean birth stories were not explained by changes in fear or self-efficacy.

**Conclusions:**

Childbirth preferences in nulligravid women can be significantly influenced by vicarious experiences. For stories about vaginal birth, the influence of birth stories on women’s fear and self-efficacy expectancy are partly responsible for this influence. The findings highlight the importance of monitoring bias in vicarious experiences, and may inform novel strategies to promote healthy childbirth.

**Supplementary Information:**

The online version contains supplementary material available at 10.1186/s12884-021-03944-w.

## Background

One in three women in the United States and in Australia give birth by caesarean, despite recommendations from the World Health Organisation (WHO) that a rate between 10 and 15% is optimal [1, 2]. A caesarean birth rate between 10 and 19% does not necessarily confer any protection against maternal or infant mortality, and a rate above 19% is clearly linked to *higher* maternal and neonatal mortality [3].

Caesarean birth has undeniable benefits in high-risk pregnancies, but its over-use has adverse outcomes on physical, emotional and mental health for both mothers and babies, and avoidable increased financial costs for the healthcare system [4–7]. Increases in pre-labour (planned) caesarean birth have contributed to an overall increase in caesarean birth that is not explained by an increase in clinical risk factors [8, 9]. Maternal request for planned caesarean birth has been suggested as one explanation [10]. Women’s childbirth preferences develop before their first pregnancy, and caesarean birth preference in nulligravid (never pregnant) women already surpasses the WHO recommended rate [11–14]. Caesarean birth has become increasingly normalised in modern cultures that pathologise childbirth as an illness necessitating medical management [15]. Even women who express a preference for vaginal birth view childbirth as a medical process requiring specialist medical expertise and intervention rather than a natural, non-pathologised process [11]. Childbirth preference for caesarean section in nulligravid women is lower in countries such as Iceland (7.6%) where the public healthcare system approaches childbirth as a natural process, than in Australia (18.4%), where a two-tiered system of private and public care that is predominantly obstetric-led favours a more medicalised view of childbirth [14, 16]. Women’s pre-childbearing preferences for caesarean delivery are likely to be influenced by social and cultural factors, as well as psychological factors that explain how these influences affect women’s specific preferences for childbirth.

### Factors influencing childbirth preferences

Childbirth self-efficacy and childbirth fear are the most frequently reported psychological factors associated with childbirth preference. Childbirth self-efficacy is defined as the belief that one is able to cope with the process of labour and vaginal childbirth [17]. Childbirth fear is defined as the fear of vaginal birth and encompasses four dimensions: fear of pain, fear of harm to the baby, fear of complications and fear of bodily damage [18]. Carlsson et al. [19] found that nulliparous (never birthed) women in their third trimester of pregnancy with the lowest self-efficacy reported higher levels of childbirth fear and higher intention of using epidural analgesia than women with higher self-efficacy. In another study, Lowe [17] found nulligravid women with lower levels of childbirth self-efficacy were more likely to plan for a caesarean birth than those with higher level of childbirth self-efficacy. These findings were reproduced in a large Australian study by Schwartz et al. [20] in which, regardless of parity, self-efficacy was significantly related to birth preference, and intentions for caesarean birth increased as self-efficacy decreased.

### Theory of self-efficacy

According to Bandura [21], self-efficacy is the product of outcome expectancy and self-efficacy expectancy. Outcome expectancy is the belief that a behaviour will lead to a given outcome while self-efficacy expectancy is the belief that one is able to perform the required behaviour in adverse situations [21]. For vaginal childbirth, outcome expectancy can be defined as the belief that a behaviour will help to cope with the process, while self-efficacy expectancy is the belief that one is able to carry out the behaviour during the process. Self-efficacy determines willingness to use coping behaviour, to what extent, and for how long [22].

Self-efficacy is influenced by four factors, in order of importance: direct experience, vicarious experience, verbal persuasion, and physiological states [21]. For nulligravid women, who are unlikely to have been exposed to any direct experience, vicarious experience is potentially the most influential factor [17]. Hauck et al. [12] found childbirth attitudes in nulligravid women, such as attitudes towards obstetric technology, physical cost of childbirth or women’s ability to choose a how to birth, were informed by vicarious experiences in the form of birth stories from family, friends, and media. Vicarious experience also influences childbirth fear. Nulligravid women who have witnessed an actual birth reported lower childbirth fear, particularly those who witnessed a home birth when compared with a hospital birth [23].

Bandura’s theory of self-efficacy provides a link between vicarious experience in nulligravid women, self-efficacy, and childbirth fear. But while self-efficacy and childbirth fear have been showed to be related to childbirth preference [20], there is very little research on the psychological processes underpinning these relationships. The already high preference for caesarean birth in nulligravid women indicates a need to understand the development of childbirth self-efficacy and fear before a woman is pregnant, by which time childbirth preferences are already established.

### Birth stories

Transmission of birth stories has evolved with the development of new technologies, and women have new levels of access to birth stories via multimedia platforms [24]. In 406 nulliparous women, Carlsson et al. [19] found 97% reported hearing birth stories, with mothers and friends as the most commonly cited sources. Although the study did not specifically ask about exposure to birth stories via social media, 80% of women reported looking for information about childbirth on this platform. In 382 nulliparous women, Amyx et al. [24] found that other women who had birthed were rated the third most important source of influence only after healthcare practitioners and prenatal classes, neither of which are relevant sources of exposure for nulligravid women. In a qualitative study in nulliparous women, birth stories were cited as the most helpful source of information by 71% of women [25]. For 45% of women, choice of childbirth was established before getting pregnant, and they were more likely to select information congruent with their choice during their pregnancy.

Representations of birth in the media and in intergenerational birth stories tend to over-represent negative or emergency events [23]. Human tendency to react more strongly to, and be more emotionally affected by, negative than positive events, may mean that negative childbirth narratives have a stronger effect on self-efficacy and fear than positive narratives, regardless of the type of birth. Although narratives that are aligned with one’s prevailing views may be perceived as more credible [23, 26], those that follow a culturally predictable pattern may also have less significance [27]. The relative influence of both negative and positive vicarious experiences for alternative types of birth on childbirth preferences remains unknown.

### The current study

This study aimed to experimentally investigate how variations in type of birth (vaginal/caesarean) and storyteller evaluation of the birth experience (positive/negative) in birth stories influenced nulligravid women’s childbirth self-efficacy, childbirth fear, and preferences for childbirth.

Based on findings from qualitative research showing that women identified birth stories as a factor influencing childbirth attitudes in nulligravid women [12], it was hypothesised that exposure to birth stories would provoke a shift in childbirth preference. Since women who express a preference towards caesarean birth report birth stories as focused on negative vaginal experience and positive caesarean birth experiences [26], it was expected that positive caesarean birth and negative vaginal birth stories would lead to an increased preference for caesarean birth. It was then intuitively presumed that positive vaginal birth and negative caesarean birth stories would lead to an increased preference for vaginal birth. The mediating effect of childbirth self-efficacy and childbirth fear in explaining changes in childbirth preference associated with the experimental manipulations was also investigated.

## Methods

### Participants

Participants were 426 nulligravid women recruited from the community through social media posts, social media advertisement, and recruitment emails from 6th August to 16th September 2018. Women were eligible to participate if they were over 18 years old, had no children but intended to have children in the future, and were not currently pregnant. The number of participants required ranged from 158 to detect a medium effect size, to 967 for a small effect size [28]. This study received approval by the QUT Human Research Ethics Committee on 25th July 2018 (Approval Number 1800000649).

### Design

A 2 (type of birth) by 2 (storyteller evaluation) experimental design was used. Type of birth was either vaginal birth or caesarean birth and storyteller evaluation was either positive or negative. Participants were randomly allocated to one of the four conditions. In each condition, participants were presented with a series of (three) individual birth stories that were consistent with the experimental manipulation for that condition. The primary outcome was change in women’s childbirth preferences following exposure. Responses were measured via self-report prior to, and directly after, exposure to the birth stories to assess changes in the primary dependent variable (childbirth preference) and potential mediators (childbirth self-efficacy and childbirth fear).

Type of birth (vaginal/caesarean) was manipulated with a clear statement by the storyteller and by scenarios congruent with the birth method. Stories began with a statement indicating the type of birth (e.g., “My vaginal birth …” ). In both scenarios, type of birth was recounted as a ‘planned experience’, and the recount included experiences of hospital arrival, steps of surgery in caesarean birth stories, and steps of labour onset, contractions, and pushing in vaginal birth stories. All stories consistently reported delivery of a healthy baby and the baby’s gender varied across the three stories within each condition.

Storyteller evaluation was manipulated in several ways. An explicit statement of negative or positive evaluation of the type of birth was included at the beginning and the end of the stories (e.g., “it was the best decision ever!” or “it ended up being the worst decision ever!”). Themes reported as influential in satisfaction with childbirth (predictability, medicalisation, medical staff support, atmosphere, pain, fear, bonding with the baby, and pleasantness of admission and discharge) were manipulated to align with the type of birth. For example, bonding with the baby was varied to express positive (e.g., “He was placed on my chest and left with me for a while”) or negative (e.g., “He was briefly placed on my chest before they whisked him away to check him up”) evaluation of the experience. More detailed description of the story compositions is provided in Table S1 in the supplementary materials.

To control for potential biases between stories, story elements were matched as closely as possible both within and between conditions. All birth stories recounted birth in hospital and described a minimum two-day length of stay. Wording of the stories was kept as similar as possible and use of emotional language was minimised to the extent that they retained realness and relatability. Presence of a partner was unspecified to prevent unwanted bias associated with this variable between conditions. Birth stories between conditions were matched on approximate word length, formatting, and layout and readability using the Flesch reading ease and the Flesch-Kincaid grade level scores provided in Microsoft Word 2016. The grade level was kept at the primary school level to maximise comprehension.

### Procedure and material

A novel, online survey was created for the purpose of this study using Qualtrics Version July 2018 (Qualtrics, Provo, UT). Participants first read a study information sheet and, upon providing consent, completed screening questions. Eligible participants continued through the survey, while ineligible participants were thanked for their time and excluded from further participation.

After completing baseline measures of childbirth preferences, fear of childbirth and childbirth self-efficacy, participants were randomly allocated to view the series of birth stories. After indicating that they read the stories, participants completed the repeat measures and manipulation checks (described below in Measures) and demographic questions. Participants were only able to proceed to the following item after providing an answer and were only able to edit their answer *within* each survey section (e.g., they could not edit pre-exposure responses once they had progressed beyond that section). After completion, participants were directed to a debriefing statement, the birth stories for all conditions, and information for assistance if needed. After submitting their survey, participants could provide their email address (stored separately from survey) to enter a draw to win a prize and to nominate to receive the study findings.

### Measures

#### Childbirth preference

Participants were asked, “How would you give birth?” and used a slider to indicate their childbirth preference, with anchors being vaginal birth and caesarean birth. To prevent response primacy bias, direction of anchors was randomised. Independent of presentation, the slider converted to a score from 0 (caesarean birth) to 100 (vaginal birth). Participants were also asked “If you had to make a choice now, indicate which birth method you would plan to have” with the option to choose vaginal birth or caesarean birth. Again, response options were presented in random order. The continuous variable was used to allow more sensitive detection of changes pre- to post-exposure, while the dichotomous variable enabled a more ecologically valid measure of pre-exposure childbirth preferences.

#### Childbirth fear

Childbirth fear was measured using an adapted version of the Childbirth Fear Prior to Pregnancy (CFPP) [18]. This 10-item scale has high internal consistency (*α* = 0.87). However, two items were found to be double-barrelled, and were each separated into two. The final scale had 12 items (e.g., “I feel I will not be able to handle the pain of childbirth”). Participants responded on a 6-point Likert scale (1 = ‘strongly agree’ to 6 = ‘strongly disagree’). The mean score was calculated by averaging scores across all items and reversed so higher scores reflected higher fear. The four items obtained from the original double-barrelled items were weighted at 0.5. Excellent internal consistency was found for the CFPP at both times (T1: *α* = 0.90; T2: *α* = 0.92).

#### Childbirth self-efficacy

Childbirth self-efficacy was measured using an adapted version of the Childbirth Self-efficacy Inventory (CBSEI) [17] . Participants were asked to evaluate the usefulness of each of 15 behaviours (e.g., “Get ready for each contraction”), on a scale from 0 (not at all helpful) to 10 (very helpful). Participants then indicated how certain they were of their capacity to use each behaviour on a scale from 0 (not at all certain) to 10 (completely sure). As the original inventory was designed for women who were pregnant or had given birth, the introductory statement was adapted so participants were asked to imagine their future childbirth experience. Childbirth was defined as “the process of labour (experiencing contractions in your uterus that will allow your baby to be born) and the birth of your baby (when the baby emerges from the uterus into the outside world through the vagina)”. Mean scores were calculated for each scale by averaging scores across all items. Excellent internal consistency was found at both time for CBSEI outcome expectancy (T1: *α* = 0.90; T2: *α* = 0.94) and CBSEI self-efficacy expectancy (T1: *α* = 0.92; T2: *α* = 0.94).

### Manipulation checks

To assess the effectiveness of experimental manipulations, participants were asked to identify the type of birth described in the stories (vaginal or caesarean), the storyteller evaluation (positive or negative), and what type of birth they believed the storytellers would choose for their next birth (vaginal or planned caesarean).

### Demographic information

Participants were asked to confirm they were female, not pregnant and had never given birth. Other demographic items included age, country of residence, marital status, education level, and employment status. Participants could also indicate whether they identified with any cultural group or ethnicity.

### Analytical approach

All analyses were conducted using IBM SPSS version 25. As responses to all items except demographics were mandatory, missing data were only expected due to participant withdrawal. Participants who provided answers for the dependent variable and mediators pre- and post-exposure to the birth stories were retained for analyses. Chi-square tests of independence were performed to assess differences in employment, education, and marital status between conditions. Univariate between groups analysis of variance (ANOVA) was conducted to assess differences in age between conditions. To assess internal validity of the continuous childbirth preference measure, the pre-exposure measure was recoded as a dichotomous variable, with scores from 0 to 49 classified as a preference for caesarean birth and scores from 50 to 100 as a preference for vaginal birth. The recoded variable was compared to the dichotomous variable in the overall participant sample and by condition.

The effect of birth stories on childbirth preference was assessed controlling for childbirth preference prior to exposure. A two-way between groups analysis of covariance (ANCOVA) was performed with post-exposure childbirth preference as the dependent variable, pre-exposure childbirth preference as a covariate, and type of birth and storyteller evaluation as fixed factors. Pairwise comparisons were used to follow-up significant interactions. To determine the effect of birth stories between positive vaginal and negative caesarean birth stories and between positive caesarean and negative vaginal birth stories, a one-way between groups ANCOVA was carried out with post-exposure childbirth preference as the dependent variable, pre-exposure childbirth preference as a covariate, and experimental condition as a fixed factor. The experimental condition variable was created by coding positive vaginal, negative vaginal, positive caesarean and negative caesarean birth stories as independent conditions.

A series of two-way between groups ANCOVAs were conducted to assess differences between experimental groups in pre-test measures for the dependent variable and mediators. After confirmation of no pre-exposure differences between conditions, changes in scores for the CFPP scale and the CBSEI outcome and self-efficacy scales were computed by subtracting the pre-exposure score from the post-exposure score. Changes in scores were used for mediation analyses to avoid issues with linearity.

Hierarchical regression was executed to determine mediating effects of change in childbirth fear, and childbirth self-efficacy expectancy and outcome expectancy, by assessing whether the relationship between condition and childbirth preference was attenuated with addition of the mediators to the model [29]**.** Due to the multicategorical independent variable, mediation analyses could only compare one condition to the other three. Three multiple hierarchical regressions were run using dummy coding for alternative reference conditions (positive vaginal, negative vaginal, and positive caesarean). At stage one of each regression, dummy coded variables were entered with the pre-exposure measure for the independent variable. At stage two, change in childbirth fear, childbirth self-efficacy, and outcome expectancy were added. Significance of the indirect effect of the mediator was assessed by bootstrap evaluation of confidence intervals using the PROCESS macro version 2.16.3 [30]**.** Confidence intervals were estimated using corrected bias bootstrapping set at 5000 samples as recommended for mediation analysis with multicategorical variables [30]**.** Assumptions of hierarchical regression, including linearity, multicollinearity and homoscedasticity were checked in accordance with Tabachnick [31]**.**

## Results

### Participants

**Fig. 1:**
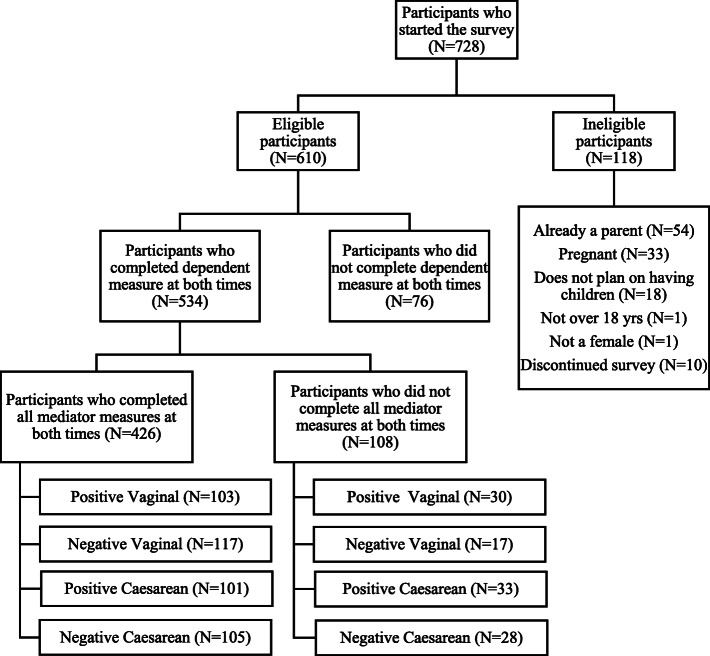
Flow Chart of Final Participant Sample

Overall, 728 participants started the survey, and 426 participants were retained for analyses (see Fig. 1).

All participants but one were Australian residents and most did not identify with any cultural group or ethnicity. About one third of participants were married, slightly less than half worked full time, and about two thirds had completed university education. The average age of the sample was 25.65 years old (age range = 18–42 years). There was no significant difference between experimental conditions in age, *F*(3,422) = 0.48, *p* = 0.698; nor in the distribution across employment, education, and marital status categories (see Table 1).

**Table 1:** Summary of Demographic Information by Conditions

	Vaginal birth stories		Caesarean birth stories			Test of group difference
	Positive	Negative	Positive	Negative	Overall	
Demographic factors	N (%)	N (%)	N (%)	N (%)	N (%)	
Country of residence						
Australia	103 (100%)	117 (100%)	100 (99%)	105 (100%)	425 (99.8%)	χ^2^(6,*N* = 425) = 3.83, *p* = 0.699^a^
Other	0 (0.0%)	0 (0.0%)	1 (1.0%)	0 (0.0%)	1 (0.2%)	
Identified with a cultural or ethnic group						
No	91 (88.3%)	96 (82.1%)	85 (84.2%)	89 (84.8%)	361 (84.7%)	χ^2^(6,*N* = 425) = 3.83, *p* = 0.699
Yes	8 (7.8%)	17 (14.5%)	14 (13.9%)	12 (11.4%)	51 (12%)	
Marital status						
Married	39 (37.9%)	34 (29.1%)	26 (25.7%)	26 (24.8%)	125 (29.3%)	χ^2^(9,*N* = 425) = 8.13, *p* = 0.521
Single	32 (31.1%)	37 (31.6%)	41 (40.6%)	40 (38.1%)	150 (35.2%)	
Other	32 (31.1%)	44 (37.6%)	33 (32.7%)	38 (36.2%)	147 (34.5%)	
Education status						
Year 12 or below	20 (19.4%)	32 (27.4%)	24 (23.8%)	25 (23.8%)	101 (23.7%)	χ^2^(9,*N* = 423) = 6.43, *p* = 0.697
Trade/Diploma	11 (10.7%)	9 (7.7%)	10 (9.9%)	14 (13.3%)	44 (10.3%)	
University	72 (69.9%)	74 (63.2%)	67 (66.3%)	65 (61.9%)	278 (65.3%)	
Employment status						
Full time	50 (48.5%)	48 (41%)	49 (48.5%)	45 (42.9%)	192 (45.1%)	χ^2^(12,*N* = 422) = 12.69, *p* = 0.392
Part time/Casual	21 (20.4%)	32 (27.4%)	28 (27.7%)	26 (24.8%)	107 (25.1%)	
Studying	31 (30.1%)	32 (27.4%)	20 (19.8%)	31 (29.5%)	114 (26.8%)	

*Note.* ^a^ Due to the small number of counts in 4 of the cells for this variable, the likelihood ratio was reported as chi-square

### Manipulation checks

Most participants correctly identified the type of birth and whether the storytellers had a positive or negative birth experience (97–100%). When asked about the future type of birth storytellers were likely to have, most participants identified the same method for stories where the storytellers were positive and the alternative method where storytellers were negative. Detailed data are presented in Table S2 in the supplementary materials.

### Preliminary analyses

The caesarean birth preference rate was 18.3% for the dichotomous measure and 18.8% using the childbirth preference measure recoded as a dichotomous measure. No differences in childbirth preference prior to exposure were found between conditions for either variables (see Table S3 in the supplementary materials), and the repartition of preference for vaginal and caesarean birth were similar for both measures within conditions. Therefore, the continuous measure of childbirth preference was used.

Those who preferred caesarean birth scored significantly higher for childbirth fear and significantly lower for both outcome expectancy and self-efficacy expectancy than those who preferred vaginal birth (Table 2).

**Table 2:** Expectancy by Preference Pre-Exposure

	Childbirth preference pre-exposure		
Variables	Vaginal birth	Caesarean birth	Condition difference
Childbirth fear	3.93 (0.94)	4.79 (0.78)	t(133) = 8.53^***^
Outcome expectancy	6.63 (1.36)	5.32 (1.80)	t(98) = 6.03^***^
Self-efficacy expectancy	5.82 (1.51)	4.54 (1.78)	t(103) = 5.88^***^

*Note.* Independent t-test statistic reported for the equal variance not assumed. ^***^p < 0.001

### Influence of birth stories on childbirth preference

**Fig. 2:**
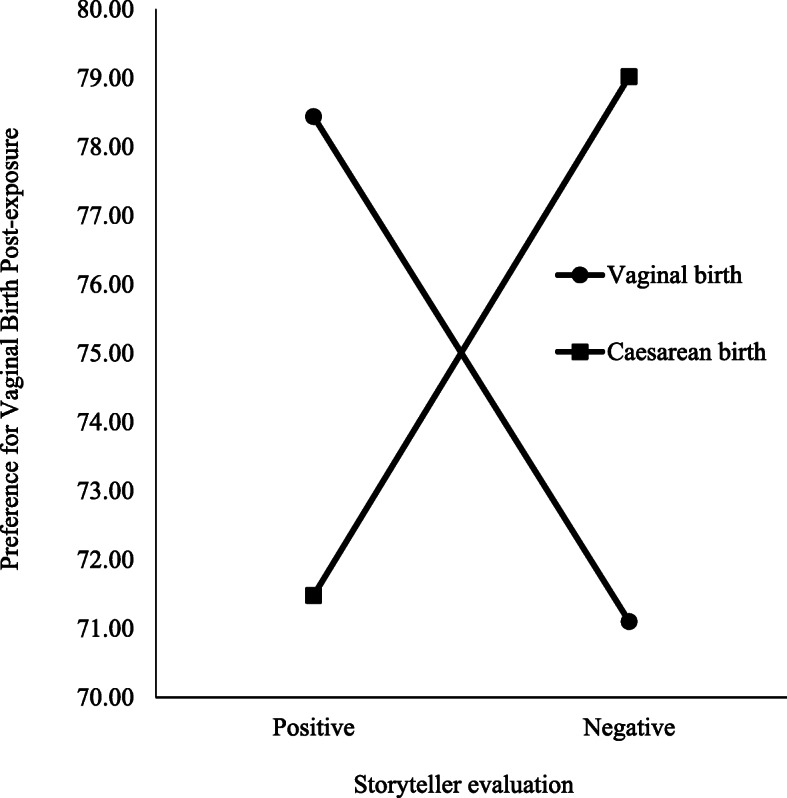
Childbirth Preference Post-Exposure by Conditions

Non-homogeneity of variance was detected for the childbirth preference measure post-exposure, and therefore alpha was set to 0.025 [32]. The ANCOVA revealed no main effect of type of birth, *F*(1,421) = 0.184, *p* = 0.668, or evaluation, *F*(1,421) = 0.01, *p* = 0.931, on childbirth preference post-exposure. However, the interaction between type of birth and storyteller evaluation was significant, *F*(1,421) = 44.78, *p* < 0.001, *η*^2^ = 0.10 (see Fig. 2).

Pairwise comparisons with Bonferroni adjustment indicated a significant difference (*p* < 0.001) in childbirth preference between the positive vaginal (*M* = 78.44, 97.5 % *CI* [75.89, 80.99]) and negative vaginal conditions (*M* = 71.10, 97.5 % *CI* [68.71, 73.49]), Cohen’s d = 0.64. Birth stories of positive vaginal experiences led to increased preference towards vaginal birth and stories of negative vaginal experiences resulted in increased preference towards caesarean birth. Pairwise comparisons indicated a significant difference (*p* < 0.001) in childbirth preference between the positive caesarean condition (*M* = 71.48, 97.5 % *CI* [68.91, 74.05]) and the negative caesarean condition (*M* = 79.02, 97.5 % *CI* [76.49, 81.43]), Cohen’s d = 0.66. Birth stories of positive caesarean experiences led to increased preference towards caesarean birth, while birth stories of negative caesarean experiences led to increased preference towards vaginal birth.

Comparisons revealed a significant difference (*p* < 0.001) in change in childbirth preference between the positive vaginal condition (*M* = 78.44, 97.5 % *CI* [75.89, 80.99]) and positive caesarean conditions (*M* = 71.48, 97.5 % *CI* [68.91, 74.05]), Cohen’s d = 0.61. Birth stories of positive vaginal experiences led to an increased preference towards vaginal birth while birth stories of positive caesarean experiences led to increased preference towards caesarean birth. Similarly, there was a significant difference (*p* < 0.001) in change in childbirth preference between the negative caesarean (*M* = 79.02, 97.5 % *CI* [76.49, 81.43]) and negative vaginal conditions (*M* = 71.10, 97.5 % *CI* [68.71, 73.49]), Cohen’s d = 0.69. Birth stories of negatively evaluated caesarean experiences led to an increased preference towards vaginal birth while birth stories of negatively evaluated vaginal experiences led to an increased preference towards caesarean birth.

Univariate ANCOVA revealed a significant effect of condition on childbirth preference, *F*(1,421) = 15.00, *p* < 0.001, *η*^2^ = 0.10. Pairwise comparisons revealed no significant differences (*p* = 1.000) in childbirth preference between the positive vaginal condition (*M* = 78.44, 97.5 % *CI* [75.89, 80.99]) and the negative caesarean condition (*M* = 79.02, 97.5 % *CI* [76.49, 81.43]), or between the positive caesarean (*M* = 71.48, 97.5 % *CI* [68.91, 74.05]) and negative vaginal conditions (M = 71.10, 97.5 % *CI* [68.71, 73.49]; *p* = 1.000). The shift in preference towards vaginal birth was similar for birth stories of positive vaginal and negative caesarean experiences, and the shift in preference towards caesarean birth was similar for positive caesarean and negative vaginal birth stories.

### Mediating effect of childbirth fear and childbirth self-efficacy

Means and standard deviations for the proposed mediators pre- and post-exposure to the birth stories are presented in Table S4 in the supplementary materials. ANCOVAs at pre-exposure revealed no significant difference between experimental conditions for childbirth preference, childbirth fear, outcome expectancy, and self-efficacy expectancy (data not shown). Therefore, change variables were used for hierarchical multiple regression and mediation analyses for childbirth preference and proposed mediators.

Multiple regression indicated that birth story condition significantly predicted post-exposure measures for childbirth fear, *R*^2^ = 0.10, *F*(3,422) = 16.46, *p* < 0.001, outcome expectancy, *R*^2^ = 0.01, *F*(3,422) = 9.21, *p* < 0.001, and self-efficacy expectancy *R*^2^ = 0.07, *F*(3,422) = 10.84, *p* < 0.001. At stage one, condition significantly predicted childbirth preference *R*^2^ = 0.10, *F*(3,422) = 14.97, *p* < 0.001 and explained 10% of the change in childbirth preference. At stage two, the addition of post-exposure measures of mediators (childbirth fear, childbirth self-efficacy expectancy, and outcome expectancy) explained an additional 3% of change in childbirth preference, ∆*R*^2^ = 0.03, ∆*F*(3,419) = 5.54, *p* = 0.001. Childbirth fear and self-efficacy expectancy contributed significantly to the model, respectively, *b* =  − 4.16, 95 % *CI*[−7.17, −1.15], *t*(419) =  − 2.72, *p* = 0.007 and *b* = 1.90, 95 % *CI* [0.36, 3.44], *t*(419) = 2.42, *p* = 0.016. Outcome expectancy did not, *b* =  − 0.17, 95 % *CI*[−1.64, 1.3], *t*(419) =  − 0.23, *p* = 0.820. As childbirth fear increased and childbirth self-efficacy expectancy decreased, preference towards caesarean birth increased. Childbirth fear was the stronger predictor of childbirth preference, and uniquely explained 2% of the variance in change in childbirth preference.

**Fig. 3:**
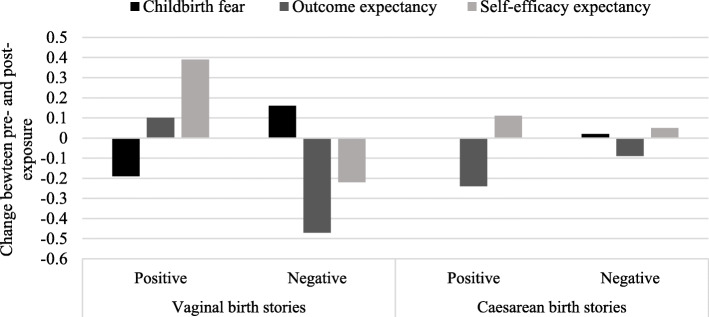
Change in the mediators after exposure to birth stories by condition

Childbirth fear and self-efficacy expectancy varied significantly by experimental condition, and significantly predicted change in childbirth preference after controlling for the effect of experimental condition. Change in mediators between pre- and post-exposure to birth stories by condition are presented in Fig. 3.

#### Influence of storyteller evaluation in vaginal birth stories

Results for each step of the mediation analysis are presented in Table 3. Evaluation in vaginal birth stories significantly predicted change in childbirth fear. In positive vaginal birth stories, childbirth fear decreased while in negative vaginal birth stories childbirth fear increased (Fig. 3). Experimental condition also significantly predicted childbirth outcome expectancy and childbirth self-efficacy expectancy (Table 3). In positive vaginal birth stories, outcome and self-efficacy expectancy slightly increased. In negative vaginal birth stories, both childbirth outcome and self-efficacy expectancy decreased (Fig. 3). As childbirth outcome expectancy did not predict the dependent variable, it was not considered further in the mediation analyses.

**Table 3:** Mediation Analysis Coefficients by Storyteller Evaluation and Type of Birth

	Path a			Path b		Indirect effect		
Change in Mediators	β	95% CI	sr^2^	β	95% CI	sr^2^	Estimate	Bootstrap Corrected bias
Storyteller Evaluation within Vaginal Birth Stories								
Childbirth fear	0.35^***^	[0.25, 0.45]	0.10	−4.16^**^	[−7.17, − 1.15]	0.02	−1.46	[− 3.58, − 0.12]
CBSEI outcome	− 0.57^***^	[− 0.79, − 0.65]	0.06	− 0.17	[−1.64, 1.3]	0.00	0.10	[−1.24, 1.07]
CBSEI self-efficacy	−0.61^***^	[− 0.82, − 0.40]	0.07	1.90^*^	[0.36, 3.44]	0.01	−1.15	[−2.74, − 0.19]
Storyteller Evaluation Within Caesarean Birth Stories								
Childbirth fear	0.02	[−0.08, 0.13]	0.00	−4.16^**^	[−7.17, −1.15]	0.02	–	–
CBSEI outcome	0.15	[−0.07, 0.38]	0.00	−0.17	[−1.64, 1.3]	0.00	–	–
CBSEI self-efficacy	− 0.06	[− 0.27, 0.16]	0.00	1.90^*^	[0.36, 3.44]	0.01	–	–
Type of Birth Within Positive Birth Stories								
Childbirth fear	0.20^***^	[0.09, 0.30]	0.03	−4.16^**^	[−7.17, − 1.15]	0.02	− 0.81	[−2.52, − 0.08]
CBSEI outcome	− 0.34^**^	[− 0.57, − 0.11]	0.02	−0.17	[− 1.64, 1.3]	0.00	0.06	[−0.70, 0.71]
CBSEI self-efficacy	0.28^*^	[−0.50, − 0.07]	0.01	1.90^*^	[0.36, 3.44]	0.01	−0.54	[−1.45, − 0.09]
Type of Birth Within Negative Birth Stories								
Childbirth fear	−0.13^**^	[−0.23,-0.03]	0.01	−4.16^**^	[−7.17, −1.15]	0.02	0.55	[0.05, 1.55]
CBSEI outcome	0.38^**^	[0.16, 0.60]	0.03	−0.17	[−1.64, 1.3]	0.00	0.07	[−0.77, 0.83]
CBSEI self-efficacy	0.27^*^	[0.06, 0.48]	0.01	1.90^*^	[0.36, 3.44]	0.01	0.51	[0.05, 1.67]

*Note.* *p < 0.05, **p < 0.01, ***p < 0.001

Evaluation in vaginal birth stories significantly predicted childbirth preference, *b* =  − 7.73, 95 % *CI*[−10.82, −4.63], *t*(422) =  − 4.91, *p* < 0.001. Exposure to positive vaginal birth stories led to an increased preference towards vaginal birth while exposure to negative vaginal birth stories led to increased preference towards caesarean birth. When mediators were added to the model, experimental condition still significantly predicted childbirth preference, *b* =  − 5.21, 95 % *CI*[−8.52, −1.91], *t*(419) =  − 3.10, *p* = 0.002, however the relationship was weakened. The indirect effects of childbirth fear and self-efficacy expectancy were significant (Table 3). Therefore, childbirth fear and childbirth self-efficacy expectancy partially mediated the effect of evaluation in vaginal birth stories on childbirth preference. In positive vaginal birth stories, increased preference towards vaginal birth was partially mediated by an increase in childbirth self-efficacy and a decrease in childbirth fear. Negative vaginal birth stories led to an increased preference towards caesarean birth, partially mediated by a decrease in childbirth self-efficacy and an increase in childbirth fear. The mediation model for exposure to vaginal birth stories is presented in Figure S1 in the supplementary materials.

#### Influence of storyteller evaluation in caesarean birth stories

Evaluation of caesarean birth did not significantly predict any of the mediators (see Fig. 3 and Table 3). Therefore, effect of the caesarean birth stories on childbirth preference was not mediated by childbirth fear, childbirth outcome, or self-efficacy expectancy.

#### Influence of type of birth within positive birth stories

Experimental condition (positive vaginal birth versus positive caesarean birth) significantly predicted childbirth fear (Table 3). In positive vaginal birth stories, childbirth fear decreased, while in positive caesarean birth stories, childbirth fear remained unchanged (Fig. 3). Condition also significantly predicted childbirth outcome expectancy, *b* =  − 0.34, 95 % *CI*[−0.57, −0.11], *t*(422) =  − 2.93, *p* = 0.004 and childbirth self-efficacy expectancy, *b* =  − 0.28, 95 % *CI*[−0.50, −0.07], *t*(422) =  − 2.55, *p* = 0.011. After exposure to positive vaginal birth stories, outcome and self-efficacy expectancy increased, although the increase in outcome expectancy appeared to be small. Exposure to positive caesarean birth stories decreased childbirth outcome expectancy and resulted in a small increase in self-efficacy expectancy (Fig. 3). Childbirth outcome expectancy did not predict the dependent variable and was not considered further.

Exposure to positive vaginal birth stories led to an increased preference towards vaginal birth while exposure to positive caesarean birth stories led to an increased preference towards caesarean birth, *b* =  − 7.20, 95 % *CI*[−10.41, −3.99], *t*(422) =  − 4.41, *p* < 0.001. When mediators were added to the model, the relationship between experimental condition and childbirth preference remained significant but weakened, *b* =  − 5.91, 95 % *CI*[−9.14, − − 2.68], *t*(419) =  − 3.59, *p* < 0.001. The indirect effects of childbirth fear and self-efficacy expectancy were significant (Table 3). Exposure to positive vaginal birth stories led to increased preference towards vaginal birth that was partially mediated by an increase in childbirth self-efficacy and a decrease in childbirth fear. Exposure to positive caesarean birth stories led to an increased preference towards caesarean birth not mediated by childbirth fear and self-efficacy. The mediation model is presented in Figure S2 in the supplementary materials.

#### Influence of type of birth within negative birth stories

Exposure to negative vaginal versus negative caesarean birth stories significantly predicted childbirth fear, childbirth outcome expectancy, and childbirth self-efficacy expectancy (Table 3). After exposure to negative vaginal birth stories, childbirth fear increased and both outcome expectancy and self-efficacy expectancy decreased (Fig. 3). In negative caesarean birth stories childbirth fear remained unchanged, and both outcome expectancy and self-efficacy expectancy remained quasi unchanged (Fig. 3). Childbirth outcome expectancy did not predict the dependent variable and was not considered further.

Negative vaginal birth stories led to increased preference towards caesarean birth while negative caesarean birth stories led to increased preference towards vaginal birth, *b* = 7.88, 95 % *CI*[4.80, 10.96], *t*(422) = 5.03, *p* < 0.001. When mediators were added, experimental condition still significantly predicted childbirth preference, *b* = 6.88, 95 % *CI*[3.79, 9.98], *t*(419) = 4.38, *p* < 0.001, however the relationship was weakened. The indirect effects of childbirth fear and self-efficacy expectancy were significant (Table 3). Exposure to negative vaginal birth stories led to increased preference towards caesarean birth that was partially mediated by a decrease in childbirth self-efficacy and an increase in childbirth fear. Exposure to negative caesarean birth stories led to an increased preference towards vaginal birth not mediated by childbirth fear or self-efficacy. The mediation model is presented in Figure S3 in the supplementary materials.

## Discussion

This study investigated the effect of birth stories (varying in type of birth and storyteller evaluation) on childbirth preference, childbirth fear, and childbirth self-efficacy in nulligravid women, and whether childbirth fear and childbirth self-efficacy (outcome expectancy and self-efficacy expectancy) mediated changes in childbirth preference. As expected, in positively evaluated birth stories, childbirth preference shifted towards the described method. In negatively evaluated stories, childbirth preference shifted away from the described method. The changes in childbirth preference significantly differed between all conditions except for between the positive vaginal and negative caesarean section birth stories, and between positive caesarean birth and negative vaginal birth stories. Childbirth fear and self-efficacy expectancy partially mediated the effect of the stories on childbirth preference. However, contrary to expectations, the outcome expectancy component of self-efficacy did not mediate the relationship between birth story exposure and childbirth preference arising from any variations in storyteller evaluation or type of birth. Additionally, childbirth self-efficacy and childbirth fear were not found to mediate the influence of caesarean birth stories on childbirth preference.

While the type of birth endorsed in the birth stories influenced the direction changes in childbirth preference, it was the combination of type of birth and storyteller evaluation that triggered the change. Neither evaluation nor type of birth had a stronger effect on childbirth preference. Even this brief exposure to birth stories influenced the childbirth preference of nulligravid women irrespective of the type of birth and the storyteller’s evaluation. As such, social and cultural influences shared through birth stories have the potential to perpetuate or minimise views of childbirth as a medicalised or non-medicalised process in the next generation of childbearing women.

It was hypothesised that childbirth self-efficacy and childbirth fear would mediate the effect of birth stories on childbirth preference. In contrast, only exposure to vaginal birth stories resulted in a change in childbirth fear and self-efficacy that was associated with changes in childbirth preference. As expected, exposure to positive vaginal birth stories led to a decrease in childbirth fear and increase in both scales of self-efficacy, however, the magnitude of the increase in outcome expectancy was small. Conversely, negative vaginal birth stories increased childbirth fear and decreased both scales of self-efficacy. In turn, childbirth fear and self-efficacy expectancy led to an increase in vaginal birth preference, but outcome expectancy did not. Therefore, change in childbirth preference generated by vaginal birth stories was partially mediated by childbirth fear and self-efficacy expectancy, but not by outcome expectancy.

Previous research reported lower levels of outcome expectancy in women who prefer caesarean birth [20]. However, no previous studies experimentally investigated the role of changes in outcome expectancy for predicting changes in childbirth preference. In the current study, outcome expectancy means appeared lower than those reported in pregnant women [17, 33]. It is possible that nulligravid women have not yet reflected on the usefulness of coping behaviours and are yet to form a defined expectancy on the subject. For vaginal birth stories, positivity mostly influenced self-efficacy expectancy. In contrast, negativity influenced both outcome and self-efficacy expectancy, but with more substantial effects on outcome expectancy. This may indicate that women are more influenced by the ability to carry out a behaviour than by its usefulness when reading a positive story. In processing negative stories, women may make an amalgam between the ability to carry a behaviour and its usefulness of a behaviour, in other words, if one cannot carry out a behaviour it must not be useful. The magnitude of the change in childbirth preference elicited by positive stories appeared to be slightly smaller than the magnitude of change elicited by negative stories. This may indicate a differential effect of outcome and self-efficacy expectancy on childbirth preference, depending on the storyteller’s evaluation of their experience.

Overall, caesarean birth stories resulted in little to no change in childbirth fear, outcome expectancy, and self-efficacy expectancy, and change in childbirth preference elicited by caesarean birth stories was not mediated by changes in childbirth fear or childbirth self-efficacy. In the current study, childbirth fear and childbirth self-efficacy were operationalised as fear of, and self-efficacy for, vaginal birth. Consequently, it is possible that the stories influenced other psychological factors specific to caesarean birth or that the influence of experiential features of caesarean birth stories were not transferred to the context of a vaginal birth experience.

This study is the first to experimentally assess the effect of birth stories on childbirth preference in nulligravid women and to consider childbirth fear and self-efficacy as explanatory psychological factors in this relationship. The final sample was of substantial size and was not limited to younger women, therefore reflecting the broader views of women that have not yet given birth. A caveat to the population representativeness was that most participating women had completed university education. Women with lower socio economic status and lower education levels are more likely to be influenced by people and media, and more likely to lack knowledge about caesarean birth indications [24]. Consequently, the effect of the birth stories on childbirth preference may be underestimated in the current sample.

Although using established scales to measure childbirth fear and self-efficacy maximised validity, reliability, and comparability with other research, the imposed survey length may have resulted in the loss of participants at the time of the post-exposure measures of childbirth fear and self-efficacy. The potential biases in participant loss that may have been associated with demographic characteristics could not be determined because these were assessed at the end of the survey. As these scales were designed specifically for vaginal birth, equivalent scales for caesarean birth are needed to assess the role of fear and self-efficacy for determining childbirth preference more accurately.

The use of a continuous measure of childbirth preference provided greater sensitivity to smaller changes in childbirth preference than an alternative dichotomous measure. However, this was at the sacrifice of ecological validity. Ultimately, there are only two options for childbirth, vaginal or caesarean birth, and thus the extent to which women’s expressed childbirth preferences reflect future childbirth decisions or outcomes remains unknown. Finally, the design of this study did not allow examination of whether the effect of the stories was short lived or long lasting, or whether the effect of stories in one category could be counteracted by another (as might be assessed in a crossover design).

The current study showed women’s childbirth preferences were affected by birth stories even though they were not yet pregnant and consequently not faced with making a deliberate choice about type of birth. This finding is striking given the incidence of positively evaluated planned caesarean birth stories in modern society may only increase in the future alongside the ever-increasing use of caesarean birth, and considering that most women who undergo planned caesarean birth are satisfied [34]. In addition, the portrayal of childbirth in the media already focuses on medicalised birth and gives little space to natural birth [35]. This is also true for birth stories, with women reporting negative vaginal experience and positive caesarean birth experiences as prominent [26]. As a result, vicarious experiences through birth stories that are most likely to be encountered by women are those increasing their fear, decreasing their self-efficacy, and consequently increasing their preference for a caesarean birth. There is growing evidence that women’s beliefs about childbirth as being a natural or medical process are associated with lower and higher levels of childbirth fear, respectively, and that women’s beliefs about birth explain the association between fear of childbirth and their birth choices [36]. The effects of vicarious experiences on beliefs about birth as a natural or medical process warrants further investigation.

Understanding the influence of vicarious experience on childbirth preference and the psychological mechanisms at play is not about attributing values to one method of childbirth over the other, but to ensure that women make decisions that are informed by unbiased information and not driven by fear or lack of self-efficacy. Women are more likely to experience post-traumatic stress symptoms when they have a childbirth that differed from their preference than when they experience the type of birth they preferred [37]. Therefore, understanding the underlying modifiable determinants of women’s preference for any method of childbirth may help prevent discordance between their preference and experience. This study highlights the need for careful educational intervention for pre-childbearing adults that aims to reduce fear and that positions childbirth as a natural process that *may* benefit from medical assistance. Future research should aim to evaluate the effect of such programs on fear, self-efficacy, and childbirth preference and to assess how birth stories can be effectively used to enhance program delivery and impact.

Globally, the rate of nulligravid women who express a preference for caesarean birth without medical indications already surpasses the WHO recommended rate for caesarean birth. Caesarean birth preference is accompanied by higher childbirth fear and lower self-efficacy. The current study showed that this preference is influenced by vicarious experience in the form of birth stories, alongside childbirth fear and self-efficacy. Negative vaginal and positive caesarean birth stories, which reflect those that women are most likely to encounter online and in social and public media, led to an increased preference for caesarean birth. This study offers preliminary evidence for the psychological mechanisms underpinning the relationship between vicarious experience and childbirth preference. It highlights the potential utility of educational programs that address childbirth fear and increase self-efficacy, and support childbirth decision-making best reflects individual needs.

## Supplementary Information

**Additional file 1.**

**Additional file 2.**

**Additional file 3.**

**Additional file 4.**

**Additional file 5.**

**Additional file 6.**

**Additional file 7.**

## Data Availability

The data set used and analysed in the current study is available from the corresponding author on reasonable request.
