# Supplementary material for: Reproducing fear: the effect of birth stories on nulligravid women’s birth preferences
Source: BMC Pregnancy Childbirth. 2021 Jun 28;21:451. doi: 10.1186/s12884-021-03944-w (PMC8240297; doi:10.1186/s12884-021-03944-w)
Supplement: Supplementary file 1 — Additional file 1. [file 12884_2021_3944_MOESM1_ESM.docx]

# Table S1

*Birth Stories Design and Composition*

**

Table S1 (Continued)
