# Supplementary material for: Reproducing fear: the effect of birth stories on nulligravid women’s birth preferences
Source: BMC Pregnancy Childbirth. 2021 Jun 28;21:451. doi: 10.1186/s12884-021-03944-w (PMC8240297; doi:10.1186/s12884-021-03944-w)
Supplement: Supplementary file 2 — Additional file 2. [file 12884_2021_3944_MOESM2_ESM.docx]

# Table S2

*Manipulation Checks*

|  | Vaginal birth stories | |  | Caesarean birth stories | |
| --- | --- | --- | --- | --- | --- |
| Manipulation checks | Positive | Negative |  | Positive | Negative |
| Type of birth in the stories |  |  |  |  |  |
| Vaginal | 100% | 100% |  | 3% | 0% |
| Caesarean | 0% | 0% |  | 97% | 100% |
| Storyteller evaluation |  |  |  |  |  |
| Vaginal | 100% | 3% |  | 100% | 1% |
| Caesarean | 0% | 97% |  | 0% | 99% |
| Choice of method for future birth |  |  |  |  |  |
| Vaginal | 99% | 11% |  | 4% | 98% |
| Caesarean | 1% | 89% |  | 96% | 2% |

*Note.* N=426
