# Supplementary material for: Reproducing fear: the effect of birth stories on nulligravid women’s birth preferences
Source: BMC Pregnancy Childbirth. 2021 Jun 28;21:451. doi: 10.1186/s12884-021-03944-w (PMC8240297; doi:10.1186/s12884-021-03944-w)
Supplement: Supplementary file 3 — Additional file 3. [file 12884_2021_3944_MOESM3_ESM.docx]

# Table S3

*Preference for Type of Birth*

|  | Experimental conditions | | | |  | Overall (N=426) | Test of difference between conditions |
| --- | --- | --- | --- | --- | --- | --- | --- |
| Pre-exposure variables | Positive Vaginal | Negative Vaginal | Positive Caesarean | Negative Caesarean |  |  |  |
|  | (N=103) | (N=117) | (N=101) | (N=105) |  |  |  |
| Childbirth preference (dichotomous) |  |  |  |  |  |  |  |
| Vaginal | 79 | 101 | 79 | 89 |  | 348 | χ^2^(3,N=426)=4.87, p=0.181 |
|  | (76.7%) | (86.3%) | (78.2%) | (84.8%) |  | (81.7%) |  |
| Caesarean | 24 | 16 | 22 | 16 |  | 78 |  |
|  | (23.3%) | (13.7%) | (21.8%) | (15.2%) |  | (18.3%) |  |
| Childbirth preference (recoded as dichotomous) |  |  |  |  |  |  |  |
| Vaginal | 76 | 101 | 81 | 88 |  | 346 | χ^2^(3,N=426)=6.26, p=0.100 |
|  | (73.8%) | (86.3%) | (80.2%) | (83.8%) |  | (81.2%) |  |
| Caesarean | 27 | 16 | 20 | 17 |  | 80 |  |
|  | (26.2%) | (13.7%) | (19.8%) | (16.2%) |  | (18.8%) |  |
