# Supplementary material for: Reproducing fear: the effect of birth stories on nulligravid women’s birth preferences
Source: BMC Pregnancy Childbirth. 2021 Jun 28;21:451. doi: 10.1186/s12884-021-03944-w (PMC8240297; doi:10.1186/s12884-021-03944-w)
Supplement: Supplementary file 4 — Additional file 4. [file 12884_2021_3944_MOESM4_ESM.docx]

# Table S4

*Means Scores of Mediators by Experimental Condition*

| Variables | Vaginal birth stories | |  | Caesarean birth stories | |
| --- | --- | --- | --- | --- | --- |
|  | Positive | Negative |  | Positive | Negative |
| Childbirth fear |  |  |  |  |  |
| Pre-exposure | 4.18 (0.92) | 4.06 (0.92) |  | 4.04 (0.98) | 4.07 (1.07) |
| Post-exposure | 3.99 (0.98) | 4.22 (1.00) |  | 4.04 (1.02) | 4.09 (1.09) |
| Change | -0.19 (0.44) | 0.16 (0.32) |  | 0.00 (0.43) | 0.02 (0.29) |
| Outcome expectancy |  |  |  |  |  |
| Pre-exposure | 6.24 (1.63) | 6.40 (1.39) |  | 6.52 (1.52) | 6.38 (1.52) |
| Post-exposure | 6.34 (1.80) | 5.93 (1.78) |  | 6.28 (1.79) | 6.29 (1.79) |
| Change | 0.10 (0.81) | -0.47 (1.06) |  | -0.24 (0.70) | -0.09 (0.70) |
| Self-efficacy expectancy |  |  |  |  |  |
| Pre-exposure | 5.41 (1.55) | 5.58 (1.60) |  | 5.61 (1.70) | 5.74 (1.71) |
| Post-exposure | 5.79 (1.73) | 5.37 (1.92) |  | 5.71 (1.87) | 5.79 (1.90) |
| Change | 0.39 (0.83) | -0.22 (0.99) |  | 0.11 (0.67) | 0.05 (0.58) |

*Note.* Standard errors are in parentheses
