# Supplementary material for: Reproducing fear: the effect of birth stories on nulligravid women’s birth preferences
Source: BMC Pregnancy Childbirth. 2021 Jun 28;21:451. doi: 10.1186/s12884-021-03944-w (PMC8240297; doi:10.1186/s12884-021-03944-w)
Supplement: Supplementary file 5 — Additional file 5. [file 12884_2021_3944_MOESM5_ESM.docx]

# Figure S1

*Mediation Model for Exposure to Vaginal Birth Stories *p<0.05, **p<0.01, ***p<0.001*


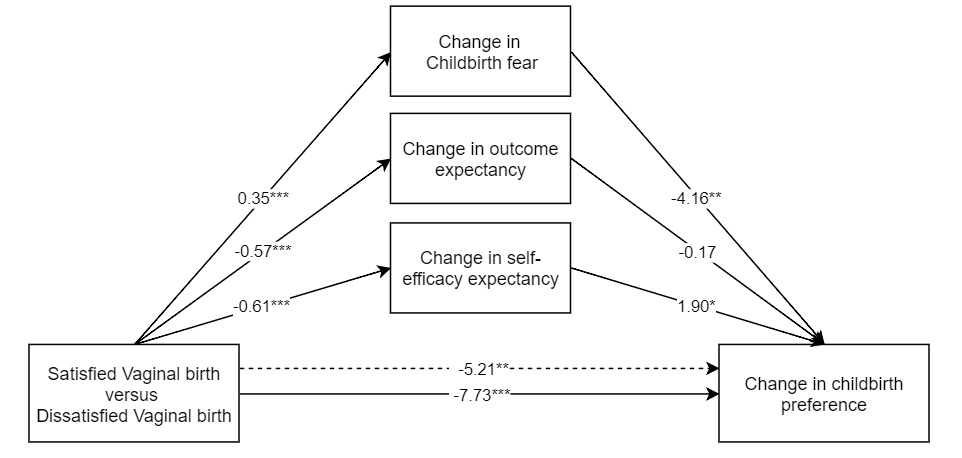


Positive vaginal birth versus

Negative vaginal birth

Change in childbirth preference
