# Supplementary material for: Reproducing fear: the effect of birth stories on nulligravid women’s birth preferences
Source: BMC Pregnancy Childbirth. 2021 Jun 28;21:451. doi: 10.1186/s12884-021-03944-w (PMC8240297; doi:10.1186/s12884-021-03944-w)
Supplement: Supplementary file 7 — Additional file 7. [file 12884_2021_3944_MOESM7_ESM.docx]

# Figure S3

*Mediation Model for Exposure to Negative Birth Stories *p<0.05, **p<0.01, ***p<0.001*


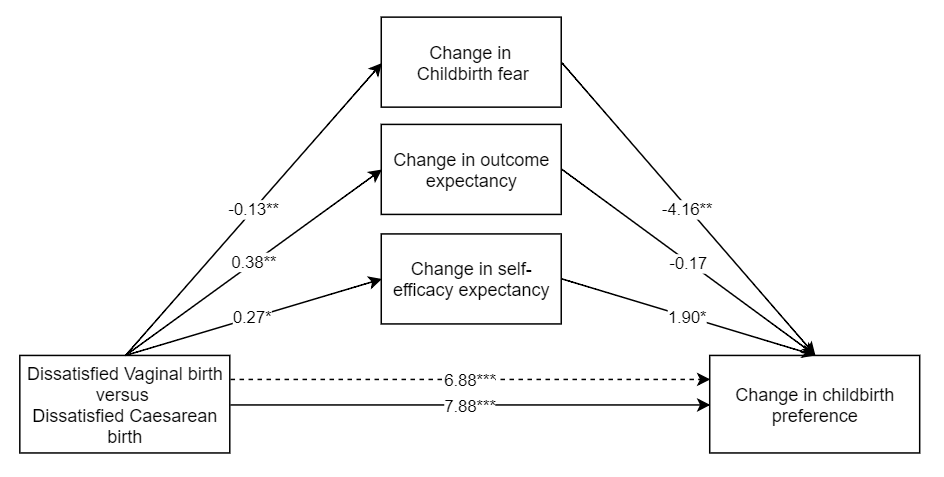


Change in childbirth preference

Negative vaginal birth

versus

Negative caesarean birth
